# Management of an Obstructed Tracheostomy in a Limited-Resource Setting

**DOI:** 10.7759/cureus.1246

**Published:** 2017-05-13

**Authors:** Julie Chiaravalli, Norman Lufesi, Elwin Shawa, Vitumbiko Nkhoma, Elaine Sigalet, Adam Dubrowski

**Affiliations:** 1 Nursing, Mzuzu Central Hospital; 2 Preventive Health Services, Ministry of Health, Malawi; 3 Intensive Care Unit, Mzuzu Central Hospital; 4 Nursing, University of Calgary; 5 Emergency Medicine, Pediatrics, Memorial University of Newfoundland

**Keywords:** nursing, developing country, medical emergency

## Abstract

Obstruction of a tracheostomy tube is a common cause of respiratory compromise in adults and pediatric patients, which can lead to a life-threatening emergency if it is not properly managed. Compromised airway patency has many potential etiologies; however, the scenario described in this technical report focuses specifically on the management of tracheostomy obstruction through the presence of a mucus plug, blood clot, or highly viscous secretions. The simulation described in this technical report was written to be conducted as an in-situ simulation within the intensive care unit (ICU) at Mzuzu Central Hospital, Malawi. The novel aspect of this report is that it depicts the integration of low-tech simulation with a deteriorating patient scenario educational methodology. This integration enables the use of affordable and sustainable simulation materials in Malawi context to deliver learning objectives that are otherwise not achievable. It was designed to train nurses, clinical officers, and nursing students from the ICU and male/female surgical wards. It can be utilized to train similar learners in other resource-poor regions of the world, as well as remote areas of the more developed countries.

## Introduction

Obstruction of a tracheostomy tube is a common cause of respiratory compromise in adults and pediatric patients, which can lead to a life-threatening emergency if it is not properly managed. Compromised airway patency has many potential etiologies but this scenario focuses specifically on the management of tracheostomy obstruction through the presence of a mucus plug, blood clot, or highly viscous secretions.

in a limited resource setting such as Mzuzu Central Hospital, Malawi, where this scenario was developed, the potential for airway compromise via tracheostomy obstruction is increased for a number of reasons. For example, only one ventilator is available for use, which leads to any new tracheostomy patient either not receiving ventilator support, or any existing patient being removed from positive pressure ventilation without the use of evidence-based weaning protocols. Suction devices are sporadically available and the suction catheters are non-sterile because they are washed and reused between patients due to lack of new equipment; these issues that are present in Malawi (and likely much of the developing world) have the potential to increase infection rates and lead to greater incidence of obstruction.

To further appreciate the challenges associated with the management of tracheostomies in this setting, it is helpful to understand the country. Malawi is one of the least developed nations in the world with the majority (84%) of its total population (18.5 million) living in rural areas and reliant on subsistence farming [1]. In 2015, the population of Mzuzu, a city in the northern part of Malawi, was reported at approximately 209,000 with 2.09 million people living in the surrounding northern region [2]. Mzuzu Central Hospital is one of four regional hospitals in Malawi and is the primary referral hospital serving the northern region. A  total of 100 patient beds makes up the intensive care unit (ICU) and male/female surgical wards. The ICU is comprised of four beds and the remaining 96 beds are shared equally between the male and female surgical wards.

This technical report describes a simulation-based teaching session we designed for nurses, nursing students, and clinical officers working in the ICU and the male/female surgical wards at Mzuzu Central Hospital who more frequently encounter patients with a tracheostomy and, therefore, have the potential to manage airway compromise. The main objective of this training exercise is to increase confidence and effective decision-making in the management of obstruction in tracheostomy patients using available resources. The simulation-based approach to the development of health professions skills in limited-resource settings has been documented by members of our group previously [3-4]. The learning objectives of this scenario were to provide clinicians with the following knowledge and skills:

1) Recognize and treat symptoms of respiratory distress and hypoxia

2) Identify an obstruction in a patient’s tracheostomy

3) Demonstrate effective decision-making and technical skills in the management of an obstructed tracheostomy

4) Perform routine tracheostomy care 

Learning objectives were scripted to align with available local resources; using technology like a stat portable chest x-ray or CT-scan are not an option and, therefore, are not part of the learning objectives or the scenario. 

For this purpose, we present a case of adapting a deteriorating patient scenario (DPS) technique to teach tracheostomy care skills to nurses and clinical officers working in the intensive care unit (ICU) and male/female surgical wards at Mzuzu Central Hospital [5]. In its original conception, DPS is a form of educational activity where the educator, typically an experienced health professional, interchangeably takes on the role of a patient and an educator. He/she can communicate vital signs, portray certain patient characteristics via acting, or cue the learners to the patient conditions in any other ways that he/she chooses. Based on these cues, the learners must treat or stabilize the portrayed patient. If the learners perform well, either as a team or as individuals, the educator will enact improvements in the patient status. If they perform poorly, the educator will enact deterioration in the patient status. Because the educator can switch roles between the patient and the educator to meet the learners’ needs, they have ample opportunity to compare their thinking to the thinking or the seasoned health professional, as well as to other learners. The session is followed by a debriefing period where learners engage in reflection on their actions. Our program utilizes a low-fidelity tracheostomy care mannequin, the Life/form® Tracheostomy Care Simulator (Nasco, Fort Atkinson, WI); therefore, we have adopted the DPS technique to include verbal prompts of changing patient conditions and deteriorations based on the learning objectives.

## Technical report

All elements supporting this simulation-based scenario, such as the educational context, inputs, processes, and expected products related to the development and implementation of this simulation case, are organized following a modified Context, Input, Process, and Product (CIPP) program development and evaluation model [6]. The management described in this technical report is closely based on one described by Gomersall, et al. [7]. For details and pictorial representations of the procedure and equipment, the readers are encouraged to access the original source.

### Context

The simulation described in this technical report was written to be conducted within the ICU at Mzuzu Central Hospital. It was designed to train nurses, clinical officers, and nursing students (hereinafter referred to as ‘learners’) from the ICU and male/female surgical wards.

### Inputs

The following equipment is required to run the simulation described here:

1. Low-fidelity tracheostomy care mannequin 2. Stethoscope 3. Oxygen therapy equipment:

- Oxygen concentrator - Trach collar, nasal cannula, and non-rebreather mask - Bag-valve mask - Oxygen saturation (SpO_2_) monitor

4. Suction equipment:

- Portable suction device - Appropriately sized suction catheter

5. Tracheostomy care equipment:

- Replacement tracheostomy tube, inner cannula, and obturator - Normal saline - Fenestrated gauze

6. Personal protective equipment:

- Gloves - Mask - Face shield

7. Printed storyboard and objective summary sheet for facilitator to follow along and provide learners with vital information as the case progresses

8. Three learners, consisting of nurses, clinical officers, and/or nursing students

9. Two simulation facilitators, who will provide supporting information, ensure adherence to the scenario case presentation, and assess individual and team performance

Sterile equipment for tracheostomy care (e.g., tracheostomy suction kit or dressing change kit) was not provided due to its unavailability in this setting.

### Process

The scenario team utilized a group of three learners made up of a combination of nurses, clinical officers, and/or nursing students. Two clinical instructors acted as facilitators and both had prior experience and training in the use of simulation in health professions education by participating in a local simulation "Train-the-Trainer" program. Before the start of the case, the facilitator established roles by selecting a simulation leader and those who will act in supporting roles. The facilitator addressed issues of confidentiality and comfort and sought an agreement to the fiction contract with all who were involved.

The scenario time was 10-15 minutes. Table 1 illustrates the step-by-step delivery of the scenario. The scenario was divided into separate phases, each of which was connected with a set of objectives, clinical findings, facilitator interactions, and expected outcomes. When actions were met, the next phase of the scenario started with a new, improved set of patient characteristics. If these actions were not met, the patient deteriorated in a pre-determined fashion.

**Table 1:** Summary of the Expected Actions Clinical findings are summarized at each step, as well as facilitator/educator inputs that may be used to prompt the learners to the appropriate expected actions.

Case Presentation			
Scenario	A 37-year-old male, three days’ post-tracheostomy placement was recently transferred from the ICU to the male surgical ward. He was involved in a road traffic accident (RTA) one week ago and has a head injury with significant facial trauma. In the report, the ICU nurse tells you his Glasgow Coma Scale (GCS) is 10. He is nonverbal (due to the tracheostomy), but he nods yes/no appropriately to questions. He opens his eyes to voice and is consistently following motor commands. In the report, the nurse gives you the following vital signs prior to transfer: pulse rate of 84 bpm, respiratory rate of 18 breaths/min (bpm), and oxygen saturation 95% on room air. ​You will now assess the patient, after arrival to your ward.		
Objective 1: Recognize and treat symptoms of respiratory distress and hypoxia			
Phase	Clinical Findings	Expected Learner Actions	Facilitator Interaction/Input
1. Initial Assessment	Noisy, labored breathing Use of accessory muscle Increased agitation Not following commands	Check SpO2 saturation Assess respiratory rate Auscultate breath sounds Provide oxygen Visibly inspect for displacement of tracheostomy Identify need for suctioning of tracheostomy Attempt to suction, using as sterile a technique as possible in this setting	Prompt for learner recall of proper positioning in patients with respiratory distress, if necessary SpO2 is now 84% on room air, despite addition of oxygen Respirations increased to a rate of 26 bpm when learner states assessing rate Report breath sounds are wet/ gurgling Tracheostomy is in place, not displaced Unable to pass suction catheter
Pathway Options…	If expected learner actions NOT MET, move to #2 Worsening Condition. If expected learner actions MET, move to #3 Ongoing Management.		
Phase	Clinical Findings	Expected Learner Actions	Facilitator Interaction/Input
2. Worsening Condition	Pulse rate 120 bpm SpO2 decreased to 80% Respiratory rate increased to 30 bpm Wet/gurgling breath sounds Use of accessory muscles Patient is increasingly lethargic/ comatose Visible secretions around stoma site	Attempt to pass suction catheter Attempt to provide positive pressure ventilation with use of bag-valve mask directly to tracheostomy Learner verbalizes that tracheostomy appears to be obstructed	Unable to pass suction catheter, due to meeting resistance Meeting resistance, not able to pass air through tracheostomy tube, via bag-valve mask
Objectives 2 & 3: Identify and manage an obstruction in a patient’s tracheostomy			
3. Ongoing Management	Clinical findings same as above, until cuff is deflated and/or inner cannula is removed Then… SpO2 increasing to 90% Pulse rate decreased to 100 bpm Respiratory rate decreased to 20 bpm Breathing appears less labored	Learner verbalizes that tracheostomy is obstructed Deflate trach cuff (if present) Obtain bag-valve mask, cover stoma, and attempt to ventilate by mouth Remove inner cannula (if present) Note obstruction in inner cannula Using sterile procedure, replace with new inner cannula	Encourage learner to take ‘next steps’ in managing obstructed tracheostomy Remind learner that O2 can be provided by other means when trach obstructed, so long as cuff is deflated, or obstructed inner cannula removed Verbalize improvement in vital signs when learner takes steps to manage obstruction
Pathway Options…	If expected learner actions NOT MET, move to #4 Worsening Condition. If expected learner actions MET, move to #5, Ongoing Management.		
Phase	Clinical Findings	Expected Learner Actions	Facilitator Interaction/Input
4. Worsening Condition	SpO2 74% Patient now in comatose state Breathing has become, slow, agonal, and grunting Cyanosis observed around lips, palms, and nailbeds Very weak palpable pulse Becomes apneic Bradycardia	Same as above in #3, Ongoing Management	Prompt learner to understand when a situation calls for emergency intervention If tracheostomy obstruction has not been identified, then instruct learner that this is an emergent situation (respiratory arrest with impending cardiac arrest) that requires patient to be taken to theater immediately or, at a minimum, to ICU for oral tracheal intubation
Objective 4: Perform routine tracheostomy care			
5. Ongoing Management	Pulse rate 90 bpm Unlabored breathing Respiratory rate 20 bpm SpO2 94% GCS is back to baseline of 10, patient is now awake and following commands	Demonstrate routine removal, cleaning, and replacement of inner cannula Suction trach using sterile technique Pre-and post-oxygenate for suctioning Demonstrate cleaning around trach site Replace fenestrated gauze Document findings and tolerance of procedure	Ask learner to demonstrate how to perform routine (daily or once per shift) tracheostomy care, now that the patient has stabilized

The first facilitator was responsible for presenting the case to the learners, describing that they have a hospitalized patient with tracheostomy recently transferred to the male surgical ward from ICU, showing early signs of respiratory distress and hypoxia. The leader was asked by the facilitator to verbalize any questions or need for clarification, and when ready, the team was asked to begin the simulation. As the simulation continued, the facilitator followed along using the information presented in Table 1, updating the simulation leader as they progressed. Once the team moved through the four phases of the scenario, the facilitator ended the simulation and a formal feedback session was started.

The second facilitator was responsible for managing the overall progression of the scenario and, through direct observation, the learning objectives. 

### Product

The scenario and teaching effectiveness were evaluated using a post-scenario survey delivered to the learners. The survey focused on learning perceptions of change in their knowledge and confidence after participating in the scenario. Additionally, the survey addressed issues of simulation effectiveness, instructional design, feedback techniques, and preparedness of the clinical instructors.

Table 2 depicts the checklists used to assess learner actions during the scenario and identify those actions, or lack thereof, that indicated performance gaps for discussion and feedback.

**Table 2:** Checklist to Assess Learner Actions Based on Learning Objectives

Objective 1: Recognize and treat symptoms of respiratory distress and hypoxia			
Step Description	Performed	Not Performed	Comments
Recognize patient in respiratory distress			
Sit patient upright			
Assess lung sounds			
Measure oxygen saturation (SpO2)			
Assess respiratory rate			
Provide oxygen (trach collar)			
Visibly inspect tracheostomy			
Verbalize need to suction tracheostomy			
Gather portable suction equipment			
Attempt to pass suction catheter			
Maintain sterile technique (as possible) to suction tracheostomy			
Attempt positive pressure ventilation using bag valve mask			
Objectives 2 & 3: Identify and manage an obstruction in a patient’s tracheostomy			
Verbalize that tracheostomy appears to be obstructed			
Deflate trach cuff (if present)			
Cover stoma, and attempt to ventilate by mouth using bag valve mask			
Remove inner cannula (if present)			
Note obstruction in inner cannula			
Using sterile technique (as possible) replace inner cannula with new			
Objective 4: Perform routine tracheostomy care			
Demonstrate routine removal, cleaning, and replacement of inner cannula			
Pre-and post-oxygenate for suctioning			
Suction tracheostomy using sterile technique			
Demonstrate cleaning around tracheostomy site			
Replace fenestrated gauze			
Document findings (stoma condition, drainage characteristics) and tolerance of procedure			

### Feedback with debriefing

A feedback session of 20-30 minutes followed the scenario and was used to discuss learner thoughts and actions, as well as best practice related to observed performance gaps. 

**Figure 1:**
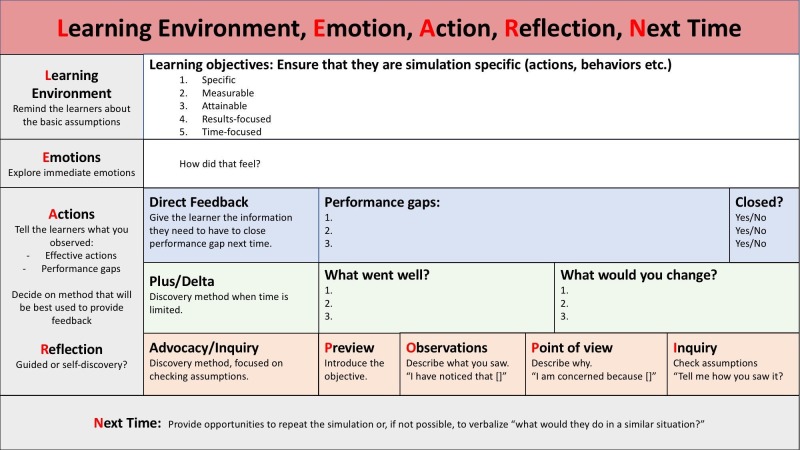
LEARN Framework

During this session, we used the Learning Environment, Emotion, Action, Reflection, Next Time (LEARN) framework (Figure 1). The LEARN framework was developed to help facilitators organize an effective feedback session [8]. According to the framework, the learners were asked to leave the simulation environment for few minutes and to refrain from discussing the scenario until prompted by the facilitator. The rationale behind this strategy is to allow facilitators an opportunity to consult with each other about the observed performance gaps and formulate their feedback plan following the LEARN steps. 

As illustrated in Figure 1, in step L (learning environment), the learners revisited the learning objectives in light of the observed performance gaps. In step E (emotions), the facilitators asked the learners to express any emotions associated with the simulation. In steps A and R (actions and reflection), one of the following feedback techniques was used:

- Direct feedback to close the gap in knowledge/skill/behavior, - plus/delta to explore the concept further as a group, - advocacy and inquiry to try and explore thoughts/thinking that led to actions so feedback can be more focused on closing the performance gap and probably more meaningful to the learner.

These different approaches were used interchangeably or in tandem within the same feedback session.

At the end of the discussion, learners were asked to articulate one thing they learned in the education session and this was used to evaluate the match between participant learning and the learning objectives. Lastly, learners were given a second opportunity to manage the scenario so they could experiment with learning and leave feeling confident about their management.

## Discussion

The aim of this simulation was to educate learners in the recognition and management of a patient in respiratory distress due to an obstructed tracheostomy, and how to perform routine tracheostomy care in a resource-limited setting, such as Malawi. This scenario was written to train nurses, nursing students, and/or clinical officers in this setting. After completion of this scenario, the learner should have increased confidence and competence in performing routine tracheostomy care and have the skills to recognize and treat potential complications that may arise in patients with tracheostomy.  

In the limited-resource setting of Mzuzu, patients receiving tracheostomy number, on average, 15 to 20 patients per calendar year. Because tracheostomy placement is a relatively infrequent occurrence and complications can lead to a life-threatening condition, it is essential to train novice practitioners using simulation to teach problem-solving skills and crisis management [9].

Caring for patients with conditions that are encountered infrequently and require more advanced training and interventions can be intimidating to novice practitioners; therefore, training through simulation is an effective tool for increasing confidence and technical skills required to care for these patients and to recognize and manage potentially life-threatening complications.

## Conclusions

Simulation-based education offers numerous advantages in both educational and health delivery systems. However, in developing countries, there are logistical and financial barriers to its use. The purpose of this technical report was to provide educators with a cost-efficient and locally developed simulation teaching scenario.

In more developed countries, teaching in a simulated setting utilizes highly realistic and computerized mannequins, which can display many patient characteristics, such as vital signs, breathing patterns, and even cyanosis. This type of simulation is typically referred to as high-fidelity simulation. Patient characteristics are adjusted by an educator, who is not present in the simulated environment most of the time but is removed and observes the learners’ actions through either a one-way viewing window or specialized audio-video equipment. Through such indirect observations, the educator determines if the learners are or are not performing the expected actions and skills and can adjust patient characteristics to improve or deteriorate the patient’s status. This is done with a computer and specialized software that runs the mannequin and the adjacent vital sign simulator – both critical parts of the simulation.

In the developing countries, such as Malawi, the use of sophisticated simulation equipment, at this point, is impossible. The costs associated with the purchase and operation costs are prohibitive, the expertise to use the software has not been developed, and the help needed to troubleshoot and maintain these pieces of equipment is inaccessible. We have successfully adopted a DPS model of education, which functions on similar principles as high-fidelity simulation. However, the DPS utilizes a combination of a non-computerized mannequin and an educator present in the same simulated environment. The educator's role is to interchangebly take on the role of the patient to enact certain patient characteristics, and an educator to cue learners to achieve the desired actions. 

We believe that this form of simulation may provide similar learning outcomes, and it utilizes significantly less advanced equipment, which can be afforded and sustained in developing countries. Furthermore, this integration of DPS and low-fidelity simulation may be also adopted for training in remote areas of developed countries. For example, health professionals in remote communities periodically find themselves in high acuity, low occurrence (HALO) patient encounters. For example, a family doctor serving a remote community may never need to intubate a pediatric patient. However, if the need arises, it is of the utmost importance that the skill is executed perfectly as if the doctor just finished pediatric emergency room training rotation. Using the model of simulation presented in this technical report offers a way of providing periodic training to health providers in remote areas, without the necessity of transporting and risking damage to high fidelity simulators.
